# Neuroendocrine tumor of the appendix associated with an appendiceal mucocele: an incidental discovery during breast tumor surveillance

**DOI:** 10.1093/jscr/rjaf787

**Published:** 2025-10-04

**Authors:** Yassine Hamdaoui, Mohamed El Hammouti, Zakaria Saber, Mohammed Doumar, Ayoub Kharkhach, Tariq Bouhout, Badr Serji

**Affiliations:** Faculty of Medicine and Pharmacy, Mohammed First University, BP 724 Hay Al Quods, Oujda 60000, Oriental, Morocco; Department of Surgical Oncology, Oncology Hospital, Mohammed VI University Hospital, BP 4806 Oujda Universite, Oujda 60049, Oriental, Morocco; Faculty of Medicine and Pharmacy, Mohammed First University, BP 724 Hay Al Quods, Oujda 60000, Oriental, Morocco; Department of Surgical Oncology, Oncology Hospital, Mohammed VI University Hospital, BP 4806 Oujda Universite, Oujda 60049, Oriental, Morocco; Faculty of Medicine and Pharmacy, Mohammed First University, BP 724 Hay Al Quods, Oujda 60000, Oriental, Morocco; Department of Surgical Oncology, Oncology Hospital, Mohammed VI University Hospital, BP 4806 Oujda Universite, Oujda 60049, Oriental, Morocco; Faculty of Medicine and Pharmacy, Mohammed First University, BP 724 Hay Al Quods, Oujda 60000, Oriental, Morocco; Department of Surgical Oncology, Oncology Hospital, Mohammed VI University Hospital, BP 4806 Oujda Universite, Oujda 60049, Oriental, Morocco; Faculty of Medicine and Pharmacy, Mohammed First University, BP 724 Hay Al Quods, Oujda 60000, Oriental, Morocco; Department of Surgical Oncology, Oncology Hospital, Mohammed VI University Hospital, BP 4806 Oujda Universite, Oujda 60049, Oriental, Morocco; Faculty of Medicine and Pharmacy, Mohammed First University, BP 724 Hay Al Quods, Oujda 60000, Oriental, Morocco; Department of Surgical Oncology, Oncology Hospital, Mohammed VI University Hospital, BP 4806 Oujda Universite, Oujda 60049, Oriental, Morocco; Faculty of Medicine and Pharmacy, Mohammed First University, BP 724 Hay Al Quods, Oujda 60000, Oriental, Morocco; Department of Surgical Oncology, Oncology Hospital, Mohammed VI University Hospital, BP 4806 Oujda Universite, Oujda 60049, Oriental, Morocco

**Keywords:** appendiceal neuroendocrine tumor, appendiceal mucocele, surgical oncology, breast tumor

## Abstract

Neuroendocrine tumors of the appendix are rare but clinically significant tumors, often discovered incidentally during surgical procedures or radiological investigations for other abdominal conditions. When they occur in association with an appendiceal mucocele, another uncommon entity characterized by abnormal dilation of the appendix due to mucus accumulation, these concurrent findings can present diagnostic and therapeutic challenges. Herein, we report the case of a 54-years-old female, previously diagnosed and treated for early-stage breast cancer. During routine surveillance, an abdominal ultrasound revealed a suspicious mass in the region of the appendix. The patient underwent abdominal computed tomography followed by abdominal magnetic resonance imaging, which demonstrated a heterogeneous mass in the right lower quadrant. The patient underwent an appendectomy and resection of the tumor.

## Introduction

Neuroendocrine tumors (NETs) can occur throughout the gastrointestinal tract, with the appendix being one of the most frequently affected sites, accounting for up to 20% of cases. These tumors are typically benign, slow-growing, and have a very low risk of metastasis. Appendiceal mucocele is another rare condition, characterized by mucinous distension of the appendix, which may be benign or malignant. It occurs in ~0.2%–0.5% of appendectomy specimens and is often asymptomatic or presents as chronic right lower quadrant pain. Preoperative identification is crucial to tailor the surgical approach and prevent complications such as pseudomyxoma peritonei. Imaging is essential, but definitive diagnosis relies on histological examination.

We present a rare case of a patient with an appendiceal NET associated with a mucocele, discovered incidentally during breast cancer follow-up, to highlight the diagnostic challenges of this association.

## Case presentation

**Figure 1:**
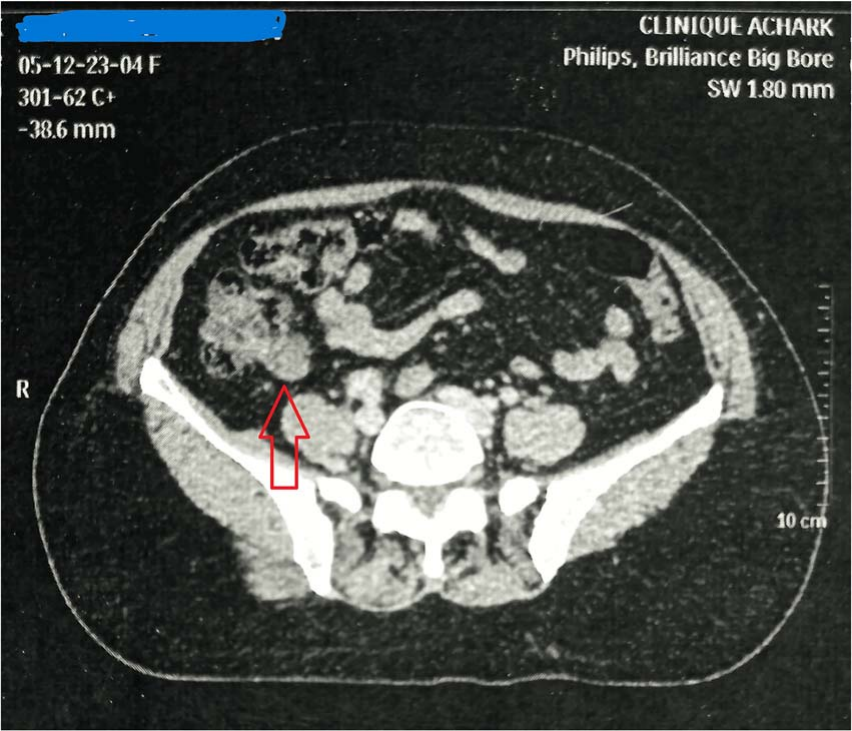
CT image showing an appendicular solido-cystic formation.

Mrs. A. H, aged 54, presented on December 2023, for further management of left breast invasive lobular carcinoma, for which she underwent lumpectomy with axillary lymph node dissection externally. The staging assessment, including abdominal computed tomography (CT) and magnetic resonance imaging (Fig. 1), revealed a mass in the right iliac fossa. On clinical examination, moderate tenderness was noted in the right iliac fossa, radiating to the left iliac fossa and right leg over the past few months. She had no complaints of vomiting or diarrhea. Her only medical history was controlled hypertension for 3 years with amlodipine and lifestyle modifications.

Clinical examination revealed significant tenderness and afirm, sensitive mass with indistinct contours and regular surface, extending into the pelvis in the right iliac fossa. Accelerated peristalsis was noted on vaginal examination, with tenderness and a mass in the right lateral fornix.

Laboratory investigations showed leukocytosis (10 000 white blood cells per cubic millimeter) and an erythrocyte sedimentation rate of 36 mm/h. Abdominal ultrasound revealed an elongated heterogeneous cystic formation in the right iliac fossa, suggestive of an appendiceal mucocele. Breast ultrasound confirmed multifocal tumor lesions retroareolarly extending from the nipple to the junction of the upper and lower quadrants of the left breast.

**Figure 2:**
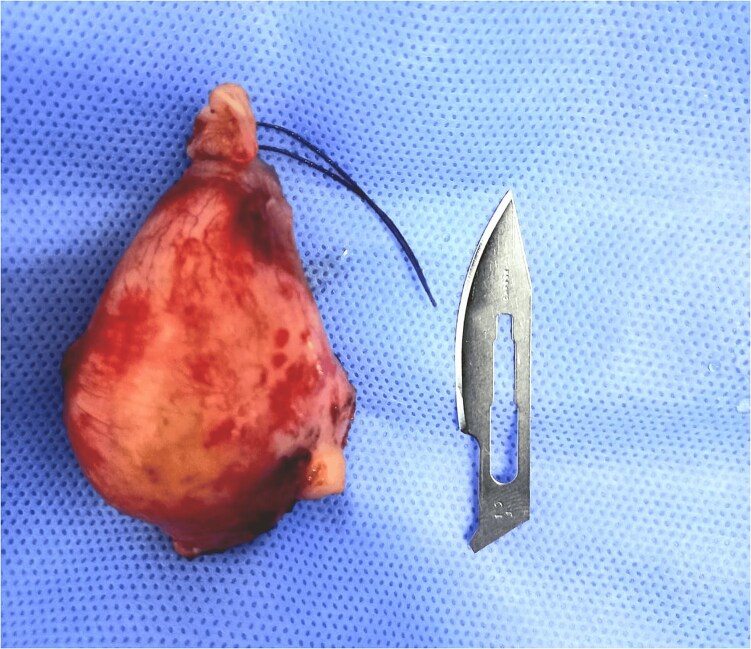
Appendectomy piece.

The patient’s case was discussed in a multidisciplinary team meeting, and the decision was made to perform simultaneous appendectomy and total mastectomy with axillary lymph node dissection (Fig. 2). A large incision revealed a well-defined oblong cystic mass measuring 5.1 cm in length and 3.4 cm in diameter, allowing for uncomplicated appendectomy. Given the intact wall of the appendiceal mucocele and cleanliness of the abdominal cavity, caution was taken to excise the cecum at the base of the appendix to prevent peritoneal cavity contamination from intraluminal secretions.

**Figure 3:**
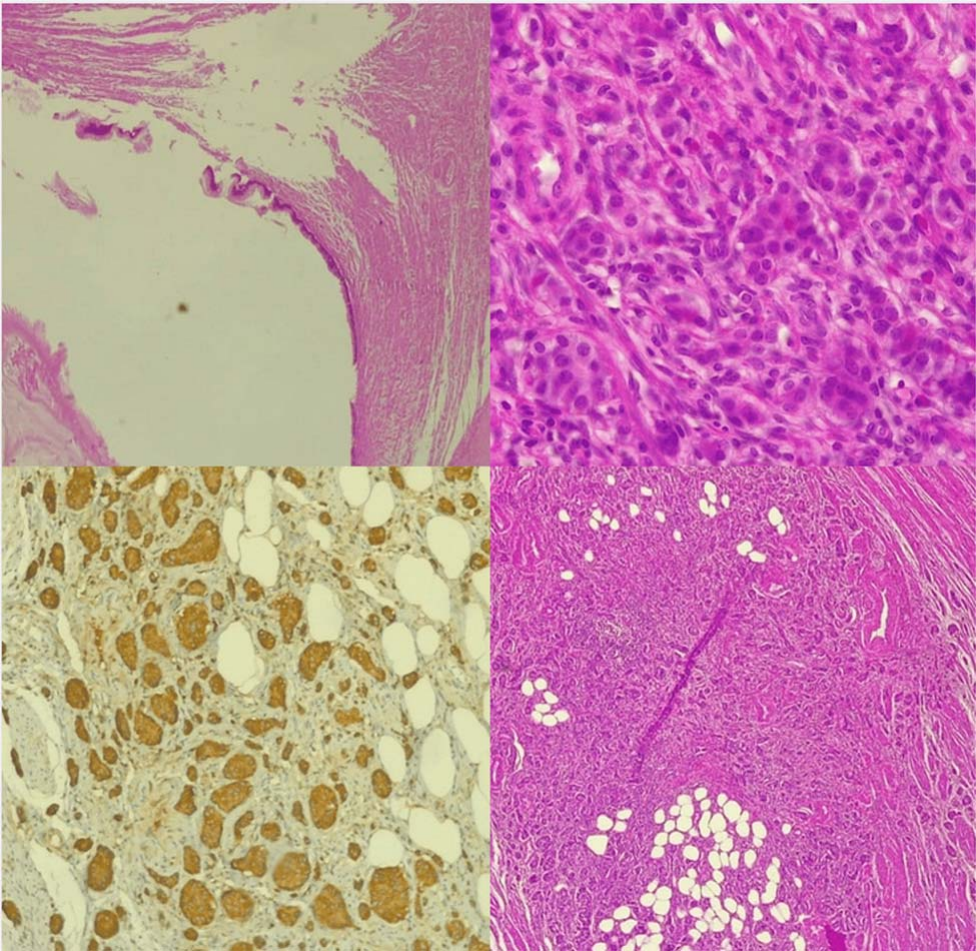
Histological image of NET of the appendix associated with an appendiceal mucocele.

Histopathological analysis confirmed a NET of the appendix associated with an appendiceal mucocele, with clear appendiceal margins. Immunohistochemical analysis showed positivity for anti-chromogranin, anti-synaptophysin, and anti-pan cytokeratin antibodies, with a Ki67 proliferation index of 1% (Fig. 3).

They are classified into three grades according to morphological differentiation and mitotic labeling index ki-67.

- Grade 1 A well-differentiated tumor or a low-grade tumor with low mitotic activity, mitotic count ˂2/2mm^2^, ki-67 < 2% and a proliferation rate that resembles a normal cell.
- Grade 2 intermediate tumors, or moderately differentiated tumors, share characteristics of grades 1 and 3 with moderate activity, mitotic count 2_20/2 mm^2^ and ki-67 3%–20%.
- Grade 3 poorly differentiated high-grading tumors have a high rate of mitotic activity and proliferation mitotic count ˃20/2 mm^2^, ki-67 > 20% .

**Figure 4:**
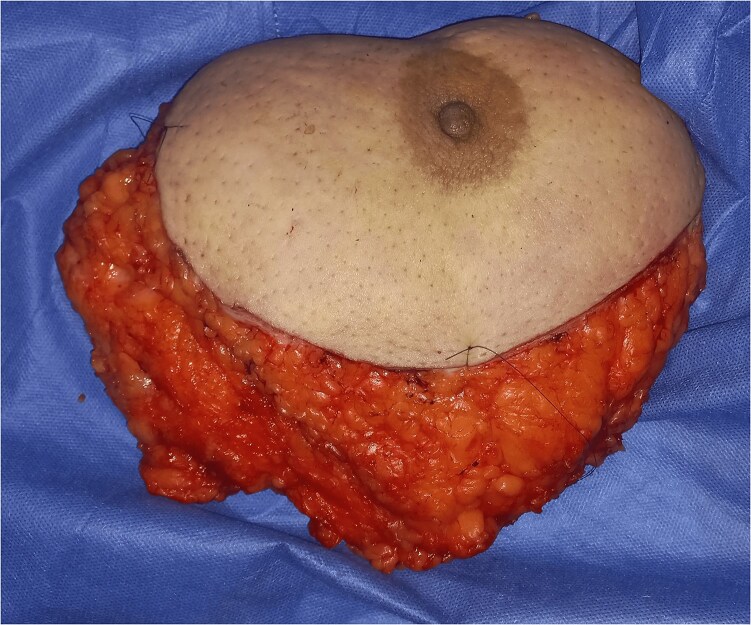
Photo of mastectomy.

Simultaneously, the patient underwent left mastectomy with axillary lymph node dissection (Fig. 4), revealing invasive carcinoma of unspecified type measuring 21 mm in diameter, Grade II SBR, classified as PT2NxMx, with negative HER2 status and a Ki67 proliferation index of 15%.

Postoperative recovery was uneventful, and the patient was discharged on the fifth postoperative day. Follow-up visits at 1 month and 2 months showed no signs of locoregional recurrence.

Based on the histopathological findings and postoperative course, adjuvant chemotherapy was initiated with regular monitoring.

**Figure 5:**
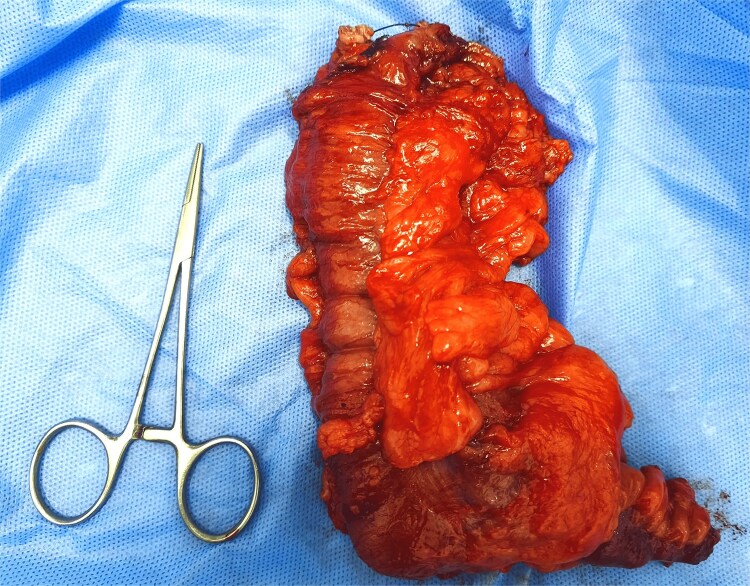
Piece of ileocecal resection.

The patient underwent an ileocecal resection (Fig. 5) with a right ileocolic anastomosis after receiving adjuvant treatment. The postoperative course was uneventful, and the patient resumed bowel transit on day 2 after surgery.

## Discussion

NETs of the appendix, also referred to as appendiceal endocrine tumors in recent literature, are rare, slow-growing tumors and represent the most common tumors of this organ [1]. They are detected with a prevalence of 0.25% to 0.85% in appendectomy patients [2], regardless of the surgical indication (annual incidence estimated at 1–2/100 000) [3]. There is a slight predominance in females, possibly reflecting a bias due to routine appendectomies performed during pelvic laparoscopic explorations. A first peak incidence is observed in the second decade for females and the third for males, with more aggressive tumors found in younger patients. A second peak incidence is noted in the eighth decade. These neoplasms were initially described by Oberndorfer a century ago as ‘carcinoid tumors’ and considered tumors derived from intraepithelial enterochromaffin cells [4]. Since then, several classifications have been proposed, considering histological architecture, neurotransmitter production, or clinical aggressiveness [5]. The World Health Organization has established a concise classification independent of tumor immunohistochemistry. These tumors generally have an excellent prognosis; however, malignant forms exist, associated with metastatic disease that can be fatal. Overall, among all NETs of the digestive tract, those affecting the appendix have the highest 5-year survival rate (almost 100% compared to 50% for stomach NETs) [6].

Appendiceal mucocele, or appendiceal mucinous cystadenoma, is defined as cystic dilation of the appendix lumen due to intraluminal accumulation of translucent, gelatinous mucinous secretions, which may involve the entire organ or a predominantly distal segment. The mucinous distension of the appendix lumen can be tumor-related or non-tumor-related, benign, or malignant [7]. First described by Rokitansky in 1842 and named by Feren in 1876 [8, 9], appendiceal mucocele is a rare condition representing 0.15%–0.6% of appendectomies [9, 10]. In our setting, we confirmed one case of appendiceal mucocele out of 874 appendectomies performed in the department over a 5-year period, yielding a frequency of 0.001%. Its frequency may be underestimated here, as not all appendectomy specimens underwent histological examination routinely. It predominantly affects adults with an average age between 50 and 60 years [11, 12], as observed in our case. However, appendiceal mucocele can also occur in children; for example, Duquenoy [13] reported this pathology in five children aged 4–13 years with cystic fibrosis. Similarly, Ekert in 1998 in France described an appendiceal mucocele in a 4.5-year-old child with cystic fibrosis [14].

Regarding gender, our case involved a female patient. The sex ratio varies across different series and seems to favor a female predominance in recent studies. However, a male predominance was noted by Souei-Mhiri in their study conducted between 1991 and 1998 in Sousse, which found six males versus four females in a series of 10 cases [15].

On CT scan, appendiceal mucocele appears as a rounded, well-defined mass at the cecal base with a thin wall and fine calcifications. Its wall may be thickened, irregular, with enhancing nodules suggestive of cystadenocarcinoma; however, there is no radiological sign to definitively confirm or exclude the malignancy of the underlying appendiceal tumor.

The management of appendiceal NETs associated with a mucocele requires not only accurate diagnosis but also careful consideration of long-term follow-up. Although small, well-differentiated NETs (<2 cm) can often be managed with simple appendectomy, larger tumors or those with mesoappendiceal or nodal involvement warrant more extensive resections such as ileocecal resection or right hemicolectomy to optimize oncological control [16]. Moreover, the presence of a mucocele introduces an additional risk of pseudomyxoma peritonei in the event of rupture, justifying meticulous surgical technique and vigilant postoperative surveillance [17]. Consequently, regular follow-up with imaging and biomarkers is recommended even in apparently low-risk cases, as this strategy improves early detection of recurrence and contributes to maintaining the excellent long-term prognosis usually associated with appendiceal NETs.

## Conclusions

Appendiceal NETs are rare and usually asymptomatic, which may lead to underdiagnosis. Despite this, their prognosis is excellent, with a 5-year survival rate exceeding 90%. While a simple appendectomy is curative in most cases, more extensive surgery may be required for larger tumors or in the presence of nodal involvement. Appendiceal mucocele, though uncommon, should be considered in cases of persistent right iliac fossa masses. Accurate preoperative diagnosis is essential to prevent rupture and pseudomyxoma peritonei. Imaging modalities such as ultrasound and CT are critical, but histopathology remains the gold standard. This case highlights the importance of clinical vigilance and a multidisciplinary approach to diagnose and manage coexisting conditions in oncology patients.
